# Ferulic Acid, an *Angelica sinensis*-Derived Polyphenol, Slows the Progression of Membranous Nephropathy in a Mouse Model

**DOI:** 10.1155/2012/161235

**Published:** 2012-07-11

**Authors:** Chao-Wen Cheng, Wen-Liang Chang, Li-Cheng Chang, Chia-Chao Wu, Yuh-Feng Lin, Jin-Shuen Chen

**Affiliations:** ^1^Graduate Institute of Clinical Medicine, College of Medicine, Taipei Medical University, No. 250 Wu-Hsing Street, Xinyi District, Taipei City 110, Taiwan; ^2^School of Pharmacy, National Defense Medical Center, No. 161, Section 6, Minquan E. Road, Neihu District, Taipei City 114, Taiwan; ^3^Division of Nephrology, Department of Internal Medicine, Tri-Service General Hospital, No. 325, Section 2, Chenggong Road, Neihu District, Taipei City 114, Taiwan; ^4^Department of Internal Medicine, Shuang Ho Hospital, Taipei Medical University, No. 291, Zhongzheng Road, Zhonghe District, New Taipei City 235, Taiwan

## Abstract

Membranous nephropathy (MN) is a leading cause of adult nephrotic syndrome but lacks adequate treatment. Different extracts of *Angelica sinensis* (AS) and one of its active compounds, ferulic acid (FA), were used to evaluate the therapeutic effects in a MN mouse model. The MN model was grouped into three subgroups: no treatment (N-T), treatment at induction of MN (Pre-T), and treatment after full-blown MN (Post-T). The results showed that the methanol (ME) layer of AS extract exhibited a therapeutic effect on MN-induced proteinuria. The ME layer-enriched compound, FA, improved the hypoalbuminemia, hyperlipidemia, and proteinuria in both Pre-T and Post-T groups. Ferulic acid also reduced the formation of oxidative protein products and increased the synthesis of antioxidant enzymes in groups Pre-T and Post-T. Regarding angiogenesis factors, the antiangiogenic factors in renal glomeruli were increased in group N-T, but, after FA treatment, only one of the antiangiogenic factors, thrombospondin-1, showed a significant decrease. Furthermore, the expression of Th2 predominant showed significant decrease in both Pre-T and Post-T groups when compared to that of N-T group. In summary, FA retarded the progression of MN, and the mechanisms involved the regulation of oxidative stresses, angiogenic and antiangiogenic factors, and attenuation of Th2 response.

## 1. Introduction

Up to one-third of uremia is caused by glomerular diseases [1, 2]. Membranous nephropathy (MN) is one of the commonest forms of glomerular disease in man and the most frequent cause of the adult idiopathic nephrotic syndrome. About 25% of cases develop progressive renal impairment often leading to end-stage renal disease [1, 3].

The definition of MN is granular deposition of IgG along the glomerular basement membrane (GBM) in the subepithelial location, indicating that immune disorder is involved. The subepithelial immunoglobulin deposition in the GBM induces glomerular capillary wall (GCW) damage, resulting in proteinuria. In other words, MN is regarded as an antigen-antibody reaction glomerulonephritis. Considering the source of antigen, up to present, some possible mechanisms have been suggested [1, 3, 4]. First, the formation of immune complexes may occur by an* in situ* mechanism in which free antigens from circulation are first deposited in the glomeruli followed by free antibodies. Second, another *in situ* mechanism is one in which antigens are from a native source, and then circulating autoantibodies react to them. The breakthrough discovery of a native antigen for MN in humans is the M-type phospholipase A2 receptor (PLA2R), leading to MN, which in this case is suggested to be an autoimmune disease [5]. Third, cationic antigens deposit in the subepithelial space since they are not restricted by the anionic charge barrier in the GBM. Once these deposits are large enough, activation of the complement system is then responsible for the membrane damage and leads to proteinuria. Based on this mechanism, we induced an MN mouse model with cBSA in our laboratory to study the pathogenesis and therapeutic approaches of MN [6, 7]. This idea was proved later by Debiec et al. suggesting that cationic bovine serum albumin (cBSA) is responsible for childhood forms of MN [8]. 

It has been suggested that the formation of oxidant stress mediates the GBM injury, leading to a fall in the glomerular filtration rate [9]. Induction of the antioxidant enzyme production can also ameliorate the severity of proteinuria in experimental MN [10]. Subsequently, the GCW injury and repair are both initiated by changing the local expression of angiogenic and antiagiogenic factors, termed angiogenesis [11, 12]. Both the angiogenic factor, vascular endothelial cell growth factor (VEGF) and antiangiogenic factors, thrombospondin-1 (TSP-1) and plasminogen kringle domain 5 (K5) are all involved in the progression of MN [13, 14]. The development of MN showed predominantly humoral Th2-mediated immune reactions [6], if attenuate this trend may also slow the damage. Inasmuch as both the formation of immune deposits in the GBM and development of GCW damage resulting in proteinuria represent the key features of MN, regimens could attenuate the status of immune reaction and the severity of glomerular capillary injury cascade, which can be applied for MN therapy. This is the goal of our study. 

Currently, several therapeutic regimens, including corticosteroids and other immunosuppressive drugs, have been studied in MN; however, their therapeutic efficacy is still unsatisfactory. New drugs or regimens with higher efficacy and fewer side effects need to be developed. The root of *Angelica sinensis* (AS), also known as “Danggui,” is a widely used herbal medicine in China for gynecological diseases [15]. Phthalides, organic acids and their esters, and polysaccharides are the main chemical components related to the bioactivities and pharmacological properties of AS. Among these, the main constituents ferulic acid (FA) and Z-ligustilide are usually chosen as marker compounds to assess the quality of AS [16, 17]. It has been reported that AS has an effect on angiogenesis *in vitro* by promoting human endothelial cells proliferation, migration, and the expression of VEGF [18]. It can also prevent oxidant injury by modulating cellular glutathione content, and this effect is in a concentration-dependent manner [19–21]. In addition, AS has also shown immunomodulatory ability *in vitro* [22]. This evidence suggests that AS has multiple pharmacological bioactivities that may provide a promising therapeutic regimen for MN.

In this study, different extract layers of AS and one of its major ingredients, FA, were used to evaluate potential therapeutic effects in an MN mouse model. Treatment with both the methanol (ME) layer of AS extract and FA slowed the progression of MN. The underlying mechanisms included the reduction of oxidative stresses, retention of angiogenic activities, and suppression of Th2 immune response.

## 2. Materials and Methods

### 2.1. MN Mouse Model

Animal studies were performed in accordance with institutional guidelines. Experiments were conducted on female eight-week-old BALB/c mice that were obtained from the National Laboratory Animal Center, Taipei, Taiwan. Experimental MN was induced by cationic bovine serum albumin (cBSA) as previously described [6]. Disease onset was checked regularly using urine dipsticks (Bayer Corp., Elkhart, IN, USA). Once dipstick showing a marked proteinuria (++++), it was labeled as full-blown MN.

### 2.2. Measurement of Urine and Blood Biochemistry

All blood and urine biochemical data, including urinary protein/urine creatinine (Up/Ucr), blood urea nitrogen (BUN), Cr, albumin (Alb), and total cholesterol (T. chol) were determined as previously described [6].

### 2.3. Evaluation of Renal Histopathology

Formalin-fixed, paraffin-embedded, and frozen sections of kidney tissue were cut and stained with hematoxylin and eosin (H&E) staining, colloidal iron, and IgG immunofluorescence stain for general histological examination as previously described [6]. 

### 2.4. Preparation of Extracts and Ferulic Acid from AS

**Figure 1:**
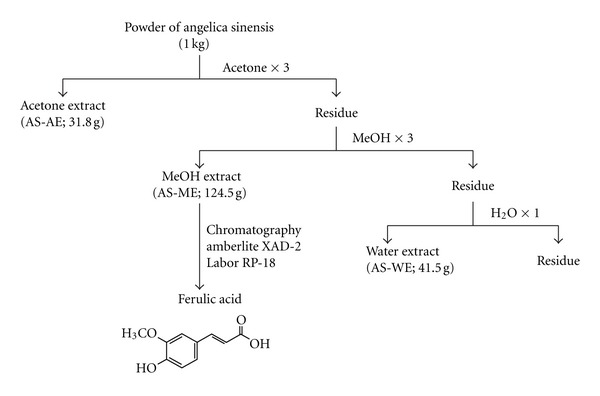
Differential extraction of *Angelica sinensis *(AS).   The powder of AS was extracted sequentially with acetone, methanol, and water. The extracts were concentrated under reduced pressure to yield an acetone extract (AS-AE), methanol extract (AS-ME), and water-soluble extract (AS-WE). The layer of AS-ME was chromatographed on an Amberlite XAD-4 column and gave 4 separated fractions, including H_2_O fraction, 30% MeOH/H_2_O fractions, 50% MeOH/H_2_O fraction, and 100% MeOH. The 100% MeOH fraction was further chromatographed on Lobar RP-18 to get purified ferulic acid.

The roots of AS (Oliv.) Diels were supplied by Chung-Yuan Co., Taipei and were identified by Dr Han-Ching Lin. A voucher herbarium specimen (NDMCP no. 920226) was deposited at the School of Pharmacy, National Defense Medical Center, Taiwan. The dried and powdered roots of AS (1.0 kg) were extracted sequentially with acetone (6 L, 3 times), methanol (6 L, 3 times), and water (4 L). The extracts were concentrated under reduced pressure to yield an acetone extract (AS-AE, 31.8 g), methanol extract (AS-ME, 124.5 g), and water-soluble extract (AS-WE, 41.5 g) (Figure 1). Each layer, including AS-ME, -AE, and -WE, was designed to treat the MN model. The preparation of AS-ME layer was dissolved in 100 *μ*L Tween 20 and then was diluted to 1 mL using PBS, and the concentrate was 60 mg/mL. The AS-AE layer was dissolved in 50 mg/mL propylene glycol and 10 mg/mL Tween 80 in distilled water. Mice were grouped for treatment with each layer and vehicle separately. The MN model was induced as previously described [6, 7]. First, mice were immunized with 0.2 mg of cBSA emulsified in an equal volume of complete Freund's adjuvant. Two weeks later, the mice began to receive intravenously injection with 13 mg/kg cBSA twice a week. At the same time, AS-ME, AE, WE, and vehicle were given to mice. Each group had at least 5 mice. Once remarkable proteinuria (dipstick ++++) occurred in the vehicle group, AS-ME, -AE, and -WE groups were assessed. Therapeutic effect was evaluated, as severity of proteinuria in treatment groups was significantly lower than that of vehicle group. Taken together, we found that the ME layer is effective for treatment of the MN mouse model. 

FA is a major active compound in the ME layer and may have therapeutic effects on MN. FA was prepared from the ME layer as follows. The AS-ME layer was chromatographed on an Amberlite XAD-4 column by elution with H_2_O, 30% MeOH/H_2_O, 50% MeOH/H_2_O, and 100% MeOH, gradually, to give 4 fractions, H_2_O fraction (80.54 g; fraction 1), 30% MeOH/H_2_O fractions (29.94 g, fraction 2), 50% MeOH/H_2_O fraction (2.70 g; fraction 3), and 100% MeOH (6.84 g; fraction 4). Fraction 4 was chromatographed on Lobar (RP-18, 70% MeOH/H_2_O) to give FA (352 mg), identified by comparison of spectroscopic data of the literatures and authentic materials (Figure 1).

The preparation of FA for MN model treatment was 150 mg dissolved in 300 *μ*L Tween 80 and 2 mL normal saline, adjusting PH to 8.5 by 1N NaOH; the final volume was 3 mL, and concentration was 50 mg/mL. Optimum dose of FA on MN treatment was determined firstly. The protocol of treatment was similar to the above description, and 2 groups were studied. The ideal dose of 30 mg/kg was chosen to evaluate the effect of FA on MN. 

### 2.5. Experimental Design

Based on the ideal dose of 30 mg/kg, four groups were designed to study the effect of FA on the MN mouse model. The first group was Pre-T; FA, 100 *μ*L, was given via intraperitoneal injection (IP) to mice at 2 weeks after immunization until the end of the experiment. The second group was Post-T, and FA, 100 *μ*L, was given via IP when the MN mice showed severe proteinuria until the end of the experiment. The third group was N-T; the vehicle, 100 *μ*L, was given via IP for MN mice. The fourth group was the NC group; normal saline, 100 *μ*L, was given via IP to control mice. The end of the experiment was 1 week after severe proteinuria development in the N-T group. At that time, the mice were euthanized, and blood, urine and kidney samples were collected for further analysis. 

### 2.6. Oxidative Stress-Related Markers

#### 2.6.1. Markers for Antioxidant Enzymes: Measurement of SOD, CAT, and GPx Activities

After perfused with PBS to remove red blood cells and clots, renal cortical tissue was homogenized in 50 mM phosphate buffer (pH 7.4). The suspensions were centrifuged at 13000 ×g and 4°C for 30 min, and then the supernatants were used for assay [23]. Total superoxide dismutase (SOD), catalase (CAT), and glutathione peroxidase (GPx) activities in renal cortical tissue were measured using the Cayman Chemical kit. Protein content was determined using the BCA protein assay.

#### 2.6.2. Markers for Oxidative Damage: Determination of Advanced Oxidation Protein Products (AOPPs), Malondialdehyde (MDA), and 8-Hydroxydeoxyguanosine (8-OHdG)

Oxidative stresses may damage all components of cell; hence we checked AOPP for protein, MDA for lipid, and 8-OHdG for DNA. AOPP concentrations were measured by a spectrophotometric assay, as described previously [24]. Briefly, 200 *μ*L of serum, urine, and renal cortical tissue were placed in a 96-well plate and mixed with 20 *μ*L acetic acid, 10 *μ*L of 1.16 *μ*M potassium iodide, and 20 *μ*L of acetic acid. AOPP concentrations were measured in a microplate reader at 340 nm and calibrated versus standard reference wells containing 200 *μ*L of chloramine-T solution (0 to 100 *μ*M). 

MDA levels were assayed for products of lipid peroxidation by monitoring thiobarbituric acid-reactive substance formation as described previously [25]. Two hundred *μ*M supernatants from homogenized renal cortical tissue or standard were mixed with 12.5 *μ*L BHT and 1250 *μ*L 0.05 M H_2_SO_4_ and incubated at room temperature for 10 mins. TBA 1500 *μ*L was added and vortexed again. The reaction mixture was then incubated at 90°C water bath for 45 mins. The tubes were then put on ice to stop the reaction. After cooling to room temperature, TBARS were extracted once with 1500 *μ*L iso butanol, and the test tubes were centrifuged at 3000 rpm for 10 mins. Two hundred *μ*L of the upper butanol phase were placed into a flat-bottom 96-well microplate, and the absorption was read at 535 nm. 

Urinary 8-OHdG levels were determined by using an ELISA kit (Japan Institute for the Control of Aging, 040914E, Japan), and the excretion of 8-OHdG was compared to concomitant urinary protein levels. 

#### 2.6.3. Marker for Oxidative Damage on Glomeruli: Tissue Myeloperoxidase (MPO) Level

Tissues were treated with paraformaldehyde and then paraffin embedded. Paraffin was removed from sections, and then the material was dehydrated. The MPO primary antibody (Pierce, PA5-16672, USA) was added and incubated at 4°C overnight. After incubation with 1 : 50 biotinylated secondary antibody (Vector laboratories, BA-1300, USA) for 40 mins, it was then treated with VECTASTAIN ABC (Vector laboratories, USA) working solution for 30 mins. The reaction was visualized by the use of DBA chromogen. The developed tissues were counterstained with hematoxylin. Sections were then observed with an optical photomicroscope. Negative controls were performed omitting primary antibodies.

### 2.7. Angiogenesis-Related Markers

#### 2.7.1. Detection of VEGF, TSP-1, and K5 Expression Level

Frozen kidney tissues were used in the IF analyses. Cryostat-cut tissue sections (4 *μ*m in thickness) were incubated with rabbit anti-TSP antibody (Novus Biologicals, CO, USA), anti-mouse K5, and goat anti-mouse VEGF antibody (R&D, MN, USA) in PBS at 4°C overnight, and then followed by 1 : 50 protein G-FITC (Zymed Laboratories, CA, USA) for 80 mins and 1 : 50 rabbit anti-goat-FITC for 90 mins (Santa Cruz, CA, USA), respectively. Negative controls were performed by omitting the primary antibody. 

#### 2.7.2. Isolation Glomeruli

The kidneys were removed and placed on a plate turned upside down on ice. We first removed the renal capsule with forceps and separated renal cortex from the kidneys and the cortical pieces were minced and pasted to a 75 *μ*m stainless-steel sieve and passed through the sieve by PBS rinsing. Then the material was isolated by further sieving through a coarse nylon sieve 100 *μ*m, then centrifuged at 1500 rpm for 5 mins at 4°C. The cell pellet was lysed by protein lysis buffer and used for Western blot analysis.

#### 2.7.3. Western Blotting

Equal amounts of protein from each sample were separated by sodium dodecyl sulfate-polyacrylamide gel electrophoresis (SDS-PAGE). The gel was equilibrated in transfer buffer at room temperature, and the protein was transferred onto PVDF (Millipore Immobilon-P, Sigma) for 2.5 h at 4°C in transfer buffer. The membranes were then blocked with 2% BSA in TBST at 4°C overnight. Next day, membranes were washed with TBST, and blots were individually incubated with antibodies against TSP-1 (abcam, ab2962, USA), K5 (R&D, MAB742, USA) or *β*-actin (SIGMA, A5441, USA) for 2 hours at room temperature, and VEGF (R&D, AF-493-NA, USA) for overnight at 4°C. Membranes were washed and followed by incubation with appropriated secondary antibodies (goat anti-rat IgG for K5, rabbit anti-goat for VEGF, goat anti-rabbit for TSP-1 and goat anti-mouse for *β*-actin) for 1 hour at room temperature. Bindings were visualized with the Western Lightning Chemiluminescence Reagent *Plus* (Perkin-Elmer Life Sciences, Boston, MA, USA) and exposed to Kodak film. 

### 2.8. Immune-Related Index

#### 2.8.1. Serum IgG1/IgG2a Level

The serum concentrations of the anti-cBSA Igs, IgG1, and IgG2a were measured using an enzyme-linked immunosorbent assay (ELISA) as previously described [26]. The IgG1 and IgG2a mouse reference sera (mouse IgG1 and IgG2a quantitation kits) were used to construct a standard curve according to the manufacturer's instructions. 

### 2.9. Data Analysis

All experiments were repeated at least three times, and data were expressed as mean ± SD. The nonparametric Kruskal-Wallis test followed by Dunn's post hoc test was used to test multiple comparisons between groups. *P* < 0.05 was considered statistically different. The Th1/Th2 immune response (serum IgG1/IgG2a) was tested by regression analysis using Statistical Product and Service Solutions (SPSS, Windows version 12.0.1C, 2000, SPSS Inc, Chicago, IL, USA).

## 3. Results

### 3.1. Protective Effect of FA-Enriched Methanol Layer of AS on MN Model

The therapeutic effects of different layers of AS extract in MN mice model were evaluated by urine dipstick analysis. Treatment with ME layer, at the dosage of 300 mg/kg per day, significantly attenuated the degree of proteinuria over those of dose 100 and 200 mg/kg. Regarding the layer of AE and WE treatment groups, there were no obvious changes in the progression of MN. Taken together, we suggest that the ME layer of AS has a protective effect on the MN mice model.

FA is an important active component in the ME layer of AS. In order to determine optimal dosage of FA in the MN model, the doses of 10, 20, 30, and 40 mg/kg were used, and evaluated by urine dipstick analysis. The therapeutic effects were a dosage dependent; however, most of the animals treated with a dose of 40 mg/kg showed weakness in addition to the attenuated severity of proteinuria. Therefore, 30 mg/kg was chosen for the following studies. 

### 3.2. FA Slowed the Progression of MN

To ascertain the protective effects of FA in the MN model, MN mice were divided into three groups by the treatment of FA, including no treatment (N-T), treatment at induction of MN (Pre-T), and treatment after full-blown MN (Post-T). As shown from the serum and urine biochemical analysis, the N-T group displayed typical MN manifestations, including hypoalbuminemia, hypercholesterolemia, and proteinuria. Treatment with FA in either Pre-T or Post-T groups resulted in significantly attenuated parameters (Table 1). 

**Figure 2:**
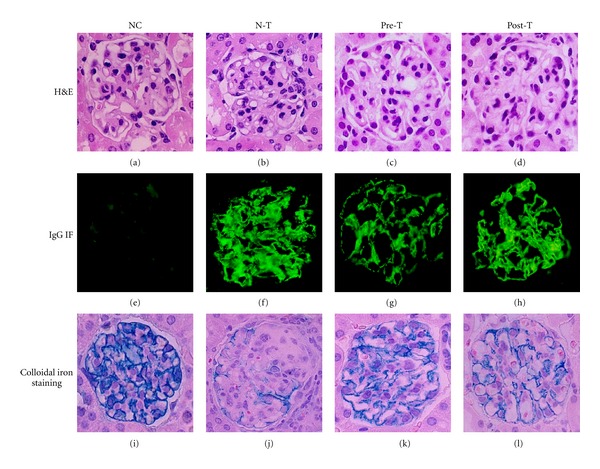
Ferulic acid attenuated the severity of renal histopathological changes in MN mouse model. At the end of the experiment, kidney samples were collected for the histopathological examination. Representative sections from renal tissue in NC ((a), (e), and (i)), N-T ((b), (f), and (j)), Pre-T ((c), (g), and (k)), and Post-T ((d), (h), and (l)) groups were performed with H&E staining ((a) through (d)), IgG immunofluorescence staining ((e) through (h)), and colloidal iron staining ((i) through (l)), original magnifications: 400x.

In microscopic examination of renal tissue sections, the N-T group exhibited the typical MN morphological patterns, namely, diffuse thickening of the GBM and no mesangial cells proliferation. The Pre-T group showed obvious improvement compared to that of the N-T group, but improvement was minor in the Post-T group (Figures 2(a), 2(b), 2(c), and 2(d)). In addition, the intensity of IgG along the GCW was also significantly decreased in the Pre-T group, but not in the Post-T group (Figures 2(e), 2(f), 2(g), and 2(h)). Colloidal iron staining indicated the existence of glomerular polyanion along the GCW. As Figures 2(i), 2(j), 2(k), and 2(l) showed, the Pre-T group was significantly attenuated for the expression of glomerular polyanion when compared with those of the N-T and Post-T groups.

### 3.3. FA Reduced Oxidative Stress in MN

**Figure 3:**
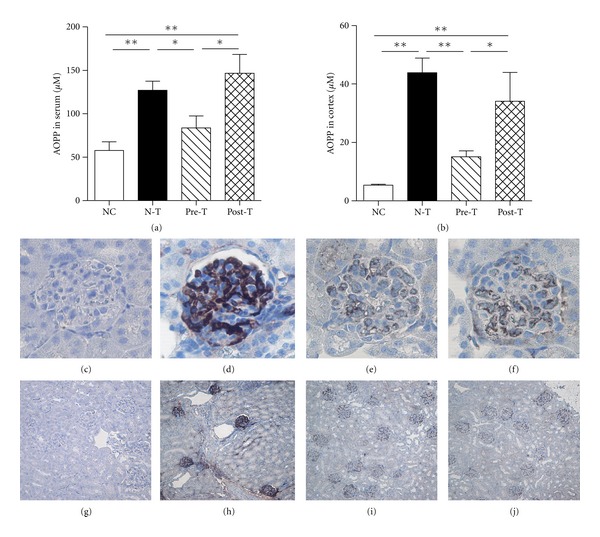
Ferulic acid reduced the level of advanced oxidation protein products (AOPPs) and myeloperoxidase (MPO) in MN mouse model. At the end of the experiment, serum and renal cortical tissues were collected, and renal cortical tissues were processed by sonicator in a ratio of per mg tissue in 5 *μ*L phosphate buffer. The formation of oxidation protein was determined by detecting the AOPPs levels in serum (a) and renal cortical tissues (b). Paraffin-embedded mouse kidney sections were examined their expression of MPO by immunohistochemical staining in NC ((c) and (g)), N-T ((d) and (h)), Pre-T ((e) and (i)) and Post-T ((f) and (j)) groups. Original magnifications: 400x ((c), (d), (e), and (f)) and 40x ((g), (h), (i), and (j)) (*P < 0.05; **P < 0.01).

**Figure 4:**
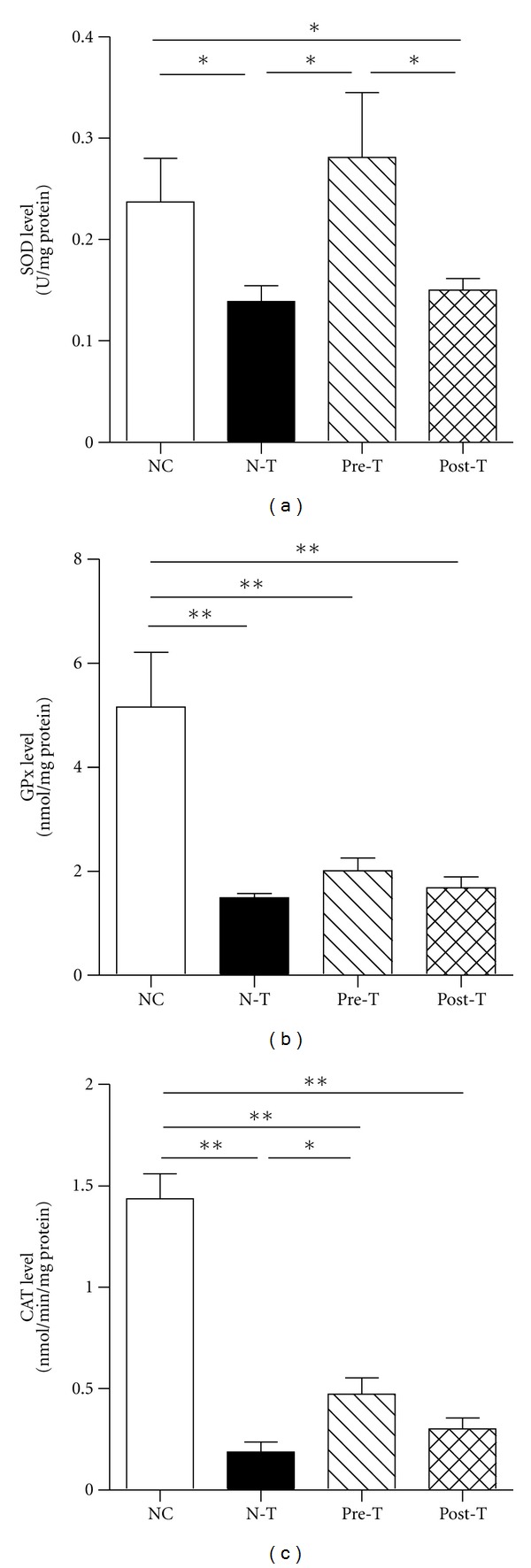
Ferulic acid increased the expression level of superoxide (SOD), glutathione (GPx), and catalase (CAT) in MN. At the end of the experiment, renal cortical tissues were processed by sonicator in a ratio of per mg tissue in 5 *μ*L phosphate buffer for detecting the level of antioxidant enzymes SOD, GPx, and CAT. The renal cortical level of SOD (a), GPx (b), and CAT (c) was determined in NC, N-T, Pre-T, and Post-T groups (*P < 0.05; **P < 0.01).

The role of oxidative stress has been shown in the pathogenesis of MN; in addition, the potent antioxidant potential of FA has also been reported. The formation of AOPP in serum and renal cortex levels was significantly diminished in the Pre-T group in comparison to that of the N-T group, but not in the Post-T group (Figures 3(a) and 3(b)). In comparison with the NC group, the expression level of MPO in the glomeruli was increased upon MN induction (N-T group; Figures 3(d) and 3(h)), and weakened in both Pre-T (Figures 3(e) and 3(i)) and Post-T groups (Figures 3(f) and 3(j)). Of note, the urinary 8-OHdG and cortical MDA levels presented no significant difference between the groups (data not shown). The antioxidant enzyme activities, SOD, CAT, and GPx, were all reduced in the N-T group; in turn, the Pre-T group exhibited improvement in the expression of antioxidant enzyme activities (Figure 4). Of note, the level of CAT also showed a recovery in the Post-T group.

### 3.4. FA Suppressed the Expression of Antiangiogenic Factor Thrombospondin-1

**Figure 5:**
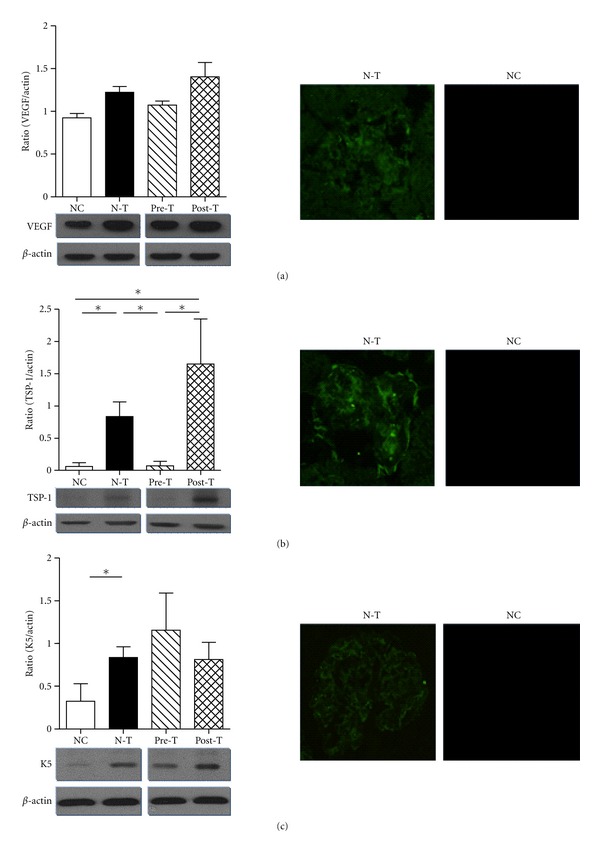
Ferulic acid regulated the differential expression of angiogenic and antiangiogenic factors in MN mouse model.  At the end of the experiment, kidney samples of different groups were harvested and freshly isolated the glomeruli according to the Materials and Methods. Total protein lysate was extracted from isolated glomeruli and separated in 10% SDS page by electrophoresis, and further blotting by anti-VEGF (a), TSP-1 (b), and K5 (c) antibodies. The bar chart represented the semiquantification level of the expression density (target/actin) (Left Panel) (*P < 0.05; **P < 0.01). Immunofluorescence staining was also undertaken to exam the expression level and location of VEGF, TSP-1, and K5 in NC and N-T, original magnifications, 400x (Right Panel).

Glomerular capillary repair is a critical process in the progression of glomerular diseases and regulated by the local balance between angiogenic and antiangiogenic factors. In the progression of MN, the glomeruli increased the expression of antiangiogenic factors (TSP-1 and K5), but the angiogenic factor (VEGF) was less affected. In the Pre-T group, the expression of TSP-1 had diminished, but not in the VEGF and K5. In addition, there were no significant changes in the Post-T group (Figure 5). These results indicate that FA had altered the process of MN-mediated glomerular capillary repair by suppressing the expression of the antiangiogenic factor, TSP-1.

### 3.5. FA Regulated the Balance of Th1/Th2 Immune Response

**Figure 6:**
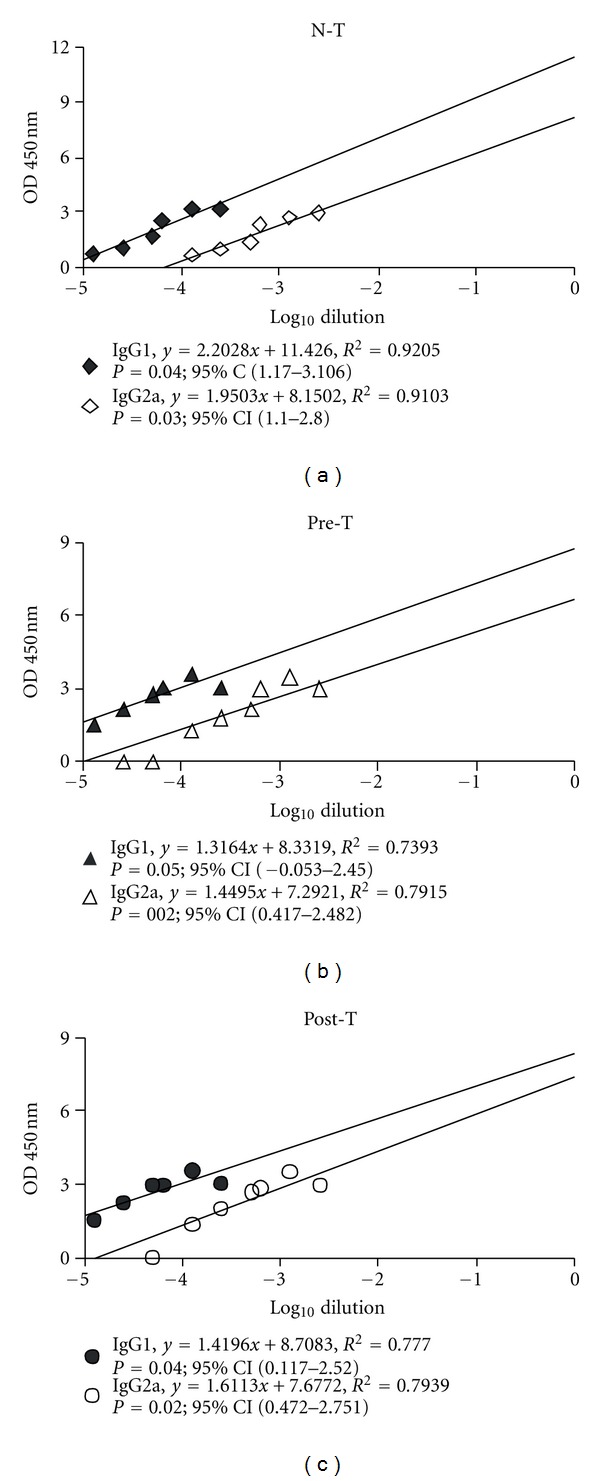
Ferulic acid changes the pattern of Th1/Th2 immune response. At the end of the experiment, serum samples were collected for determining the serum IgG1 and IgG2a level. The quantification of IgG1 and IgG2a was using a commercialized ELISA kit and analyzed in spectrometer. Data were calculated by regression analysis. Each symbol represented an individual sample and the intercept, *R* ^2^ of the regression lines, interval of confidence, and *P* value were further determined in NT (a), Pre-T (b), and Post-T (c) groups.

In our previous study, Th2 subset immune response was dominant in the development of cBSA-induced MN mouse model. After conducting the linear regression analysis by SPSS, *R* ^2^ value, interval of confidence, and *P* value were obtained. As shown in Figure 6, the intercept and *R* ^2^ of the regression lines for IgG1 were higher than those of IgG2a in the N-T, Pre-T and Post-T groups, indicating a Th2-dominant immune response in all three groups. However, the slopes of IgG1 and IgG2a among three groups showed differences. The relative ratio of slope of IgG1 between N-T and Pre-T was 1.67 (2.2028/1.3164), and that of IgG2a was 1.35 (1.9503/1.4495). Upon N-T and Post-T, the ratio of slope of IgG1 was 1.55 (2.2028/1.4196), and that on IgG2a was 1.21 (1.9503/1.6113). The decreased rate of slope in IgG1 was larger than IgG2a when comparing N-T group with Pre-T or Post-T group, suggesting that the therapeutic effect of FA on MN may involve the attenuation of the Th2 subset.

## 4. Discussion

Despite recent advances in immunosuppressive therapy, GN remains the most common disease leading to end-stage renal failure. Among them, MN is one of the most common forms of GN and the most frequent cause of adult idiopathic nephrotic syndrome. In this study, we identified the crude ME layer of AS extract as representing the protective effects in the deterioration of an MN mouse model. The AS-ME-enriched compound, FA, ameliorated MN-induced hypoalbuminemia, hyperlipidemia, and proteinuria. In renal histopathological analysis, the GBM thickening, intensity of IgG, and the glomerular polyanion in FA-treated groups, all displayed significant improvement. Pretreatment of FA in MN mouse model also showed the multiple effects, including, (1) regulation of the balance between oxidative stress and antioxidant enzyme, (2) suppression of the expression of antiangiogenic factor, and (3) mediation of the alteration of Th1/Th2 balance.

Since AS contains multiple compounds, the extracts from the differential processes of extraction methods may have different effects [15]. In our study, we used the solvents partition method in preparing the nonpolarity, moderate polarity, and strong polarity fractions in acetone, methanol, and water extracts, respectively. In this study, AS-ME showed therapeutic effects in the MN mouse model, while AS-AE and AS-WE had no effect. The layer of AS-AE having compounds with nonpolarity, such as n-butylidenephthalide, represents high cytotoxicity. Therefore, this layer is suggested as ideal for anticancer treatment [15]. The layer of AS-WE has compounds with high polarity, such as carbohydrate, inorganic acid, sugar, and amino acid; therefore, the therapeutic effects for inflammatory diseases would be minimal. The layer of AS-ME has compounds with moderate polarity, such as FA. This layer is rich in polyphenol compounds, which have antioxidant effects that are ideal for the treatment of inflammatory diseases. Here, we identified that AS-ME has the most potential therapeutic effects on the MN mouse model.

In the layer of AS-ME, we demonstrated that FA is the active compound and proved the therapeutic effects of FA in the MN mouse model. Actually, many compounds are included in AS, and the main active components are FA and ligustilide [27]. FA has been identified as having an effect on the attenuation of carbon tetrachloride-induced liver lipid peroxidation and improved the antioxidant enzymes expression [28, 29]. In STZ-induced diabetic mice model, FA can also alleviate oxidative stress and attenuate the hyperglycemic response [30]. Indeed, oxidants derived either from infiltrating leukocytes or from resident glomerular cells can mediate GBM damage and induce proteinuria, leading to a fall in the glomerular filtration rate [9]. MPO, a neutrophil cationic enzyme that localizes in the glomeruli, can react with H_2_O_2_ and halides to form highly reactive products [31]. The albuminuria and the glomerular lesions in antiglomerular basement membrane nephritis model are also highly dependent on the presence of neutrophils in the glomeruli [32]. In Figure 3, pretreatment with FA suppressed oxidative protein formation in serum and renal cortex, and the MPO expression level in the glomeruli was also diminished in both Pre-T and Post-T groups. The synthesis of antioxidant enzymes can inhibit the formation of certain oxidative free radicals, such as hydrogen peroxide, hydroxyl radical, and superoxide. The catalase-deficient mice are more susceptible to oxidant tissue injury and renal fibrosis [33]. Treatment with the SOD mimetic, tempol, can also help to preserve the glomerular capillary permeability barrier to protein in the anti-GBM rat model [34]. In this study, the MN model had showed a decreasing expression of these antioxidant enzymes, but pretreatment with FA restored the expression (Figure 4). Also, FA showed the dual ability to manipulate the formation of oxidative protein and retain the antioxidant enzymes expression, suggesting its potential application in oxidative stress-related diseases.

In addition to oxidative stress-mediated GBM injury, the regulation of angiogenic process is also involved in the pathogenesis of chronic kidney injury. The process of angiogenesis includes endothelial cell proliferation, migration, basement membrane degradation, and new lumen organization. The loss of VEGF expression and the increase in TSP-1 expression were both correlated with capillary loss and the development of glomerulosclerosis and interstitial fibrosis [35, 36]. Treatment with recombinant VEGF enhances the glomerular capillary repair and accelerates the resolution of experimentally induced glomerulonephritis [37]. Besides, in podocyte specific VEGF-deficient mice also exhibit abnormalities in the glomeruli structure and proteinuria [38]. The use of VEGF inhibitors is also associated with proteinuria in patients, suggesting that angiogenesis status is related to maintain the function of glomeruli [39]. FA has also been identified as having the ability to promote endothelial cell proliferation and angiogenesis through upregulation of VEGF [40, 41]. Although there was no significant difference in the VEGF expression level in isolated glomeruli between the NC and N-T groups, the immunofluorescence staining still displayed a lower expression tendency in the NC group (Figure 5(a)). In Figure 5(b), the glomeruli showed increasing expression of TSP-1 in the progression of the MN model. Plasminogen domain kringle 5 has previously been reported as involved in the progression of MN, as well as suppressing endothelial cell growth [7, 42]. Pretreatment with FA significantly diminished the expression of TSP-1 but did not regulate VEGF or K5 expression (Figure 5). In comparison to immunoblotting assay, isolated glomeruli had constitutively expressed VEGF in the NC group and had relatively low expression in TSP-1. Upon the induction of MN, the expression of TSP-1 was upregulated and indicated that, in the processing of MN, the induction of TSP-1 may play a detrimental role in mediating glomerular capillary repair. These findings suggested that FA may regulate the process of glomerular capillary repair by specifically reducing the glomerular TSP-1 expression; however, this specific inhibition ability was not presented in the Post-T group.

The differential subsets of helper T cells have been proposed as being involved in the pathogenesis of glomerular nephritis. The Th1-predominant immune responses are associated strongly with proliferative and crescentic forms of GN while Th2 responses are associated with membranous patterns of injury [43]. Th1 and Th2 cells can balance each other's actions, suggesting that certain strategies in modulating Th1/Th2 balance may influence the severity of disease. In development of the cBSA-induced MN mouse model, the Th2 immune response was dominant [6]. After treatment with FA, the tendency of Th2-dominant immune response was attenuated (Figure 6). Actually, several polyphenol compounds have been shown to be involved in the Th1/Th2 regulatory activity in different models though they had a different shift tendency. Chlorogenic acid and naringenin chalcone and marine red alga can all suppress pulmonary eosinophilia and Th2-type cytokine production in the ovalbumin-induced allergic asthma model [44–46]. In turn, ecklonia cava extract can decrease the production level of Th1-type cytokines, whereas increase Th2-type cytokines on murine splenocytes [47]. Tobacco polyphenols, CGA and rutin, activate IgE production *in vivo* and enhance Th2 development [48]. Here, we proved the potency of FA in inhibiting Th2-dominant immune response in MN. In addition, FA showed anti-inflammatory effects in colitis induced by dextran sulphate sodium in mice [49]. Treatment with FA can suppress the E-selectin expression and leukocyte adhesion [50]. These data indicate that FA has also immuno-regulatory properties. 

Regarding the pathogenesis of MN, the relationship among these three possible mechanisms is addressed. According to our findings, this cBSA-induced MN mouse model may present a multiple-step pathogenic cascade in the disease progression. First, the dysregulation of immune system leads to the formation of immune deposits in the GBM. Subsequently, immune deposits induce an improper balance between ROS generation and scavenging activities in mediating the GCW damage, as well as disruption of the glomerular capillary repair, resulting in proteinuria. These pathogenic cascades may also occur in glomerular injury in human MN [51]. Intervention in this process may offer therapeutic opportunities. In our study, as seen in Figures 2 and 6, treatment with FA inhibited Th2-dominant immune response, reduced the immune deposits in GBM, and maintained the integrity of GCW. This suggests that blockage of the formation of immune deposits in the GBM may also suppress the downstream pathogenic cascades and further arrested the progression of disease.

All in all, FA, the active compound of AS, can play a protective role to slow the progression of MN in the mouse model. The underlying mechanisms include its antioxidant activity, retention of angiogenesis, and modulation of immune response. FA, an herbal medication with diverse clinical pharmacological benefits, may be further applied for human MN therapy. 

## Figures and Tables

**Table 1:** Ferulic acid attenuates the severity of serum and urine parameters in MN mouse model.

		NC (*n* = 9)	N-T (*n* = 8)	Pre-T (*n* = 11)	Post-T (*n* = 12)
	Cr. (mg/dL)	0.51 ± 0.16	0.43 ± 0.06	0.47 ± 0.12	0.37 ± 0.08
Blood	T. chol. (mg/dL)	187.36 ± 24.7^∗^	447.58 ± 38.41	249.06 ± 72.33^∗^	226.37 ± 56.91^∗^
	Alb. (g/dL)	2.23 ± 0.17^∗^	1.64 ± 0.34	2.00 ± 0.19^∗^	2.08 ± 0.15^∗^

Urine	DPL (U_P_/U_Cr_)	0.47 ± 0.08^∗^	1.28 ± 0.04	0.51 ± 0.21^∗^	0.49 ± 0.15^∗^

Data are expressed as mean ± SD.

*P < 0.05 versus N-T group.

NC: normal control, N-T: no treatment, Pre-T: treatment at induction of MN, Post-T: treatment after full-blown MN, BUN: blood urea nitrogen, Cr: creatinine, T.chol: total cholesterol, Alb: albumin, and DPL (U_p_/U_cr_): daily protein loss (urine protein/urine creatinine).

## References

[1] Oliveira DBG (1998). Membranous nephropathy: an IgG4-mediated disease. *The Lancet*.

[2] Ferraz FH, Martins CG, Cavalcanti JC (2010). Profile of glomerular diseases in a public hospital of Federal District, Brazil. *Jornal Brasileiro de Nefrologia*.

[3] Massry SG, Glassock RJ (1995). *Massry & Glassock's Textbook of Nephrology*.

[4] Kerjaschki D, Neale TJ (1996). Molecular mechanisms of glomerular injury in rat experimental membranous nephropathy (Heymann nephritis). *Journal of the American Society of Nephrology*.

[5] Beck LH, Bonegio RGB, Lambeau G (2009). M-type phospholipase A2 receptor as target antigen in idiopathic membranous nephropathy. *The New England Journal of Medicine*.

[6] Wu CC, Chen JS, Lin SH, Chen A, Sytwu HK, Lin YF (2008). Experimental model of membranous nephropathy in mice: sequence of histological and biochemical events. *Laboratory Animals*.

[7] Chen JS, Hwang JC, Chang LC, Wu CC, Lin YF (2010). Attributes of antiangiogenic factor plasminogen kringle 5 in glomerulonephritis. *Archives of Pathology and Laboratory Medicine*.

[8] Debiec H, Lefeu F, Kemper MJ (2011). Early-childhood membranous nephropathy due to cationic bovine serum albumin. *The New England Journal of Medicine*.

[9] Shah SV (2006). Oxidants and iron in progressive kidney disease. *Journal of Renal Nutrition*.

[10] Wu CC, Lu KC, Chen JS (2008). HO-1 induction ameliorates experimental murine membranous nephropathy: anti-oxidative, anti-apoptotic and immunomodulatory effects. *Nephrology Dialysis Transplantation*.

[11] Maeshima Y, Makino H (2010). Angiogenesis and chronic kidney disease. *Fibrogenesis Tissue Repair*.

[12] Miyamoto K, Kitamoto Y, Tokunaga H (2004). Protective effect of vascular endothelial growth factor/vascular permeability factor 165 and 121 on glomerular endothelial cell injury in the rat. *Laboratory Investigation*.

[13] Thompson EM, Hughes J, Van Noorden S, Sharpe J, Savill J (1996). Expression of the multifunctional extracellular matrix protein thrombospondin in crescentic glomerulonephritis. *Journal of Pathology*.

[14] Schrijvers BF, Flyvbjerg A, De Vriese AS (2004). The role of vascular endothelial growth factor (VEGF) in renal pathophysiology. *Kidney International*.

[15] Cheng YL, Chang WL, Lee SC (2004). Acetone extract of *Angelica sinensis* inhibits proliferation of human cancer cells via inducing cell cycle arrest and apoptosis. *Life Sciences*.

[16] Yi L, Liang Y, Wu H, Yuan D (2009). The analysis of Radix Angelicae Sinensis (Danggui). *Journal of Chromatography A*.

[17] Zhao KJ, Dong TTX, Tu PF, Song ZH, Lo CK, Tsim KWK (2003). Molecular genetic and chemical assessment of radix Angelica (Danggui) in China. *Journal of Agricultural and Food Chemistry*.

[18] Lam HW, Lin HC, Lao SC (2008). The angiogenic effects of *Angelica sinensis* extract on HUVEC in vitro and zebrafish in vivo. *Journal of Cellular Biochemistry*.

[19] Wu SJ, Ng LT, Lin CC (2004). Antioxidant activities of some common ingredients of traditional chinese medicine, *Angelica sinensis*, *Lycium barbarum* and *Poria cocos*. *Phytotherapy Research*.

[20] Wojcikowski K, Wohlmuth H, Johnson DW, Rolfe M, Gobe G (2009). An in vitro investigation of herbs traditionally used for kidney and urinary system disorders: potential therapeutic and toxic effects. *Nephrology*.

[21] Po YC, Hoi YL, Siu AHY (2007). Dang-Gui Buxue Tang protects against oxidant injury by enhancing cellular glutathione in H9c2 cells: role of glutathione synthesis and regeneration. *Planta Medica*.

[22] Wilasrusmee C, Siddiqui J, Bruch D, Wilasrusmee S, Kittur S, Kittur DS (2002). In vitro immunomodulatory effects of herbal products. *American Surgeon*.

[23] You Y, Kim K, Yoon HG (2010). Chronic effect of ferulic acid from Pseudosasa japonica leaves on enhancing exercise activity in mice. *Phytotherapy Research*.

[24] Witko-Sarsat V, Gausson V, Nguyen AT (2003). AOPP-induced activation of human neutrophil and monocyte oxidative metabolism: a potential target for N-acetylcysteine treatment in dialysis patients. *Kidney International*.

[25] Jentzsch AM, Bachmann H, Fürst P, Biesalski HK (1996). Improved analysis of malondialdehyde in human body fluids. *Free Radical Biology and Medicine*.

[26] Chen JS, Chen A, Chang LC (2004). Mouse model of membranous nephropathy induced by cationic bovine serum albumin: antigen dose-response relations and strain differences. *Nephrology Dialysis Transplantation*.

[27] Song ZH, Ji ZN, Lo CK (2004). Chemical and biological assessment of a traditional Chinese herbal decoction prepared from radix astragali and radix angelicae sinensis: orthogonal array design to optimize the extraction of chemical constituents. *Planta Medica*.

[28] Srinivasan M, Rukkumani R, Sudheer AR, Menon VP (2005). Ferulic acid, a natural protector against carbon tetrachloride-induced toxicity. *Fundamental and Clinical Pharmacology*.

[29] Srinivasan M, Sudheer AR, Menon VP (2007). Ferulic acid: therapeutic potential through its antioxidant property. *Journal of Clinical Biochemistry and Nutrition*.

[30] Ohnishi M, Matuo T, Tsuno T (2004). Antioxidant activity and hypoglycemic effect of ferulic acid in STZ-induced diabetic mice and KK-Ay mice. *BioFactors*.

[31] Johnson RJ, Couser WG, Chi EY (1987). New mechanism for glomerular injury. Myeloperoxidase-hydrogen peroxide-halide system. *Journal of Clinical Investigation*.

[32] Schrijver G, Schalkwijk J, Robben JCM, Assmann KJM, Koene RAP (1989). Antiglomerular basement membrane nephritis in beige mice. Deficiency of leukocytic neutral proteinases prevents the induction of albuminuria in the heterologous phase. *Journal of Experimental Medicine*.

[33] Kobayashi M, Sugiyama H, Wang DH (2005). Catalase deficiency renders remnant kidneys more susceptible to oxidant tissue injury and renal fibrosis in mice. *Kidney International*.

[34] Duann P, Datta PK, Pan C, Blumberg JB, Sharma M, Lianos EA (2006). Superoxide dismutase mimetic preserves the glomerular capillary permeability barrier to protein. *Journal of Pharmacology and Experimental Therapeutics*.

[35] Kang DH, Anderson S, Kim YG (2001). Impaired angiogenesis in the aging kidney: vascular endothelial growth factor and thrombospondin-1 in renal disease. *American Journal of Kidney Diseases*.

[36] Kang DH, Joly AH, Oh SW (2001). Impaired angiogenesis in the remnant kidney model: I. Potential role of vascular endothelial growth factor and thrombospondin-1. *Journal of the American Society of Nephrology*.

[37] Masuda Y, Shimizu A, Mori T (2001). Vascular endothelial growth factor enhances glomerular capillary repair and accelerates resolution of experimentally induced glomerulonephritis. *American Journal of Pathology*.

[38] Eremina V, Sood M, Haigh J (2003). Glomerular-specific alterations of VEGF-A expression lead to distinct congenital and acquired renal diseases. *Journal of Clinical Investigation*.

[39] Eremina V, Baelde HJ, Quaggin SE (2007). Role of the VEGF-A signaling pathway in the glomerulus: evidence for crosstalk between components of the glomerular filtration barrier. *Nephron*.

[40] Wang J, Yuan Z, Zhao H (2011). Ferulic acid promotes endothelial cells proliferation through up-regulating cyclin D1 and VEGF. *Journal of Ethnopharmacology*.

[41] Lin CM, Chiu JH, Wu IH, Wang BW, Pan CM, Chen YH (2010). Ferulic acid augments angiogenesis via VEGF, PDGF and HIF-1*α*. *Journal of Nutritional Biochemistry*.

[42] Ji WR, Barrientos LG, Llinás M (1998). Selective inhibition by kringle 5 of human plasminogen on endothelial cell migration, an important process in angiogenesis. *Biochemical and Biophysical Research Communications*.

[43] Tipping PG, Kitching AR (2005). Glomerulonephritis, Th1 and Th2: what’s new?. *Clinical and Experimental Immunology*.

[44] Kim HR, Lee DM, Lee SH (2010). Chlorogenic acid suppresses pulmonary eosinophilia, IgE production, and Th2-type cytokine production in an ovalbumin-induced allergic asthma: activation of STAT-6 and JNK is inhibited by chlorogenic acid. *International Immunopharmacology*.

[45] Iwamura C, Shinoda K, Yoshimura M, Watanabe Y, Obata A, Nakayama T (2010). Naringenin chalcone suppresses allergic asthma by inhibiting the type-2 function of CD4 T cells. *Allergology International*.

[46] Jung WK, Choi I, Oh S (2009). Anti-asthmatic effect of marine red alga (*Laurencia undulata*) polyphenolic extracts in a murine model of asthma. *Food and Chemical Toxicology*.

[47] Ahn G, Hwang I, Park E (2008). Immunomodulatory effects of an enzymatic extract from Ecklonia cava on murine splenocytes. *Marine Biotechnology*.

[48] Gong J, Liu FT, Chen SS (2004). Polyphenolic antioxidants enhance IgE production. *Immunological Investigations*.

[49] Islam MS, Murata T, Fujisawa M (2008). Anti-inflammatory effects of phytosteryl ferulates in colitis induced by dextran sulphate sodium in mice. *British Journal of Pharmacology*.

[50] Wang XL, Hu XH, Lü ME, Gu ZL, Ruan CG (2005). Effects of ferulic acid on E-selectin expression in activated endothelial cell and leukocyte-endothelial cell adhesion. *Yaoxue Xuebao*.

[51] Glassock RJ (2010). The pathogenesis of idiopathic membranous nephropathy: a 50-year odyssey. *American Journal of Kidney Diseases*.

