# Corrigendum to “Ferulic Acid, an *Angelica sinensis*-Derived Polyphenol, Slows the Progression of Membranous Nephropathy in a Mouse Model”

**DOI:** 10.1155/2016/7315875

**Published:** 2016-05-24

**Authors:** Chao-Wen Cheng, Wen-Liang Chang, Li-Cheng Chang, Chia-Chao Wu, Yuh-Feng Lin, Jin-Shuen Chen

**Affiliations:** ^1^Graduate Institute of Clinical Medicine, College of Medicine, Taipei Medical University, No. 250 Wu-Hsing Street, Xinyi District, Taipei City 110, Taiwan; ^2^School of Pharmacy, National Defense Medical Center, No. 161, Section 6, Minquan E. Road, Neihu District, Taipei City 114, Taiwan; ^3^Division of Nephrology, Department of Internal Medicine, Tri-Service General Hospital, National Defense Medical Center, No. 325, Section 2, Chenggong Road, Neihu District, Taipei City 114, Taiwan; ^4^Department of Internal Medicine, Shuang Ho Hospital, Taipei Medical University, No. 291, Zhongzheng Road, Zhonghe District, New Taipei City 235, Taiwan

In the article titled “Ferulic Acid, an *Angelica sinensis*-Derived Polyphenol, Slows the Progression of Membranous Nephropathy in a Mouse Model” [1] the third affiliation was incorrect. The correct affiliations are shown above.

## References

[1] Cheng C.-W., Chang W.-L., Chang L.-C., Wu C.-C., Lin Y.-F., Chen J.-S. (2012). Ferulic acid, an *Angelica sinensis*-derived polyphenol, slows the progression of membranous nephropathy in a mouse model. *Evidence-Based Complementary and Alternative Medicine*.

